# Supplementary material for: Individual, family, school and neighborhood predictors related to different levels of physical activity in adolescents: A cross-sectional study
Source: PLoS One. 2024 Aug 23;19(8):e0304737. doi: 10.1371/journal.pone.0304737 (PMC11343401; doi:10.1371/journal.pone.0304737)
Supplement: S1 Table — V1, Steps; V2, LPA; V3, MVPA; V4, Age; V5, SitT; V6, Sleep; V7, ST; V8, Gender; V9, BF; V10, Work; V11, Commute type; V12, Consumption of alcohol; V13, Stress; V14, Loneliness; V15, Father’s education; V16, Father’s BMI; V17, Spaces for PE; V18, Extra PA class; V19, Frequency of PE classes; V20, Place to walk; V21, Walk to the bus stop; V22, Paved roads; V23, Lighting; V24, Crime; V25, Commute time to the bus stop. (DOCX) [file pone.0304737.s001.docx]

**Supplementary Table 1. Spearman’s Correlation.**

| Variables | V1 | V2 | V3 | V4 | V5 | V5 | V6 | V7 | V8 | V9 | V10 | V11 | V12 | V13 | V14 | V15 | V16 | V17 | V18 | V19 | V20 | V21 | V22 | V23 | V24 | V25 | V26 |
| --- | --- | --- | --- | --- | --- | --- | --- | --- | --- | --- | --- | --- | --- | --- | --- | --- | --- | --- | --- | --- | --- | --- | --- | --- | --- | --- | --- |
| V1 | 1.00 |  |  |  |  |  |  |  |  |  |  |  |  |  |  |  |  |  |  |  |  |  |  |  |  |  |  |
| V2 | 0.47* | 1.00 |  |  |  |  |  |  |  |  |  |  |  |  |  |  |  |  |  |  |  |  |  |  |  |  |  |
| V3 | 0.90* | 0.40* | 1.00 |  |  |  |  |  |  |  |  |  |  |  |  |  |  |  |  |  |  |  |  |  |  |  |  |
| V4 | 0.11 | 0.04 | 0.06 | 1.00 |  |  |  |  |  |  |  |  |  |  |  |  |  |  |  |  |  |  |  |  |  |  |  |
| V5 | -0.01 | -0.01 | -0.04 | 0.03 | 1.00 |  |  |  |  |  |  |  |  |  |  |  |  |  |  |  |  |  |  |  |  |  |  |
| V5 | -0.09 | -0.2* | -0.09 | -0.08 | -0.02 | 1.00 |  |  |  |  |  |  |  |  |  |  |  |  |  |  |  |  |  |  |  |  |  |
| V6 | -0.07 | -0.06 | -0.04 | 0.01 | 0.13* | -0.08 | 1.00 |  |  |  |  |  |  |  |  |  |  |  |  |  |  |  |  |  |  |  |  |
| V7 | -0.3* | -0.10 | -0.38* | -0.04 | 0.03 | 0.05 | 0.06 | 1.00 |  |  |  |  |  |  |  |  |  |  |  |  |  |  |  |  |  |  |  |
| V8 | 0.16* | 0.09 | 0.17* | 0.01 | -0.06 | -0.04 | -0.03 | -0.43* | 1.00 |  |  |  |  |  |  |  |  |  |  |  |  |  |  |  |  |  |  |
| V9 | 0.30* | 0.29* | 0.27* | 0.11 | 0.03 | -0.09 | -0.08 | -0.17* | 0.10 | 1.00 |  |  |  |  |  |  |  |  |  |  |  |  |  |  |  |  |  |
| V10 | 0.19* | -0.2* | 0.19* | 0.04 | 0.03 | 0.10 | -0.04 | -0.25* | 0.01 | 0.06 | 1.00 |  |  |  |  |  |  |  |  |  |  |  |  |  |  |  |  |
| V11 | 0.09 | 0.09 | 0.07 | 0.20* | 0.07 | 0.04 | 0.04 | -0.07 | -0.05 | 0.13* | -0.01 | 1.00 |  |  |  |  |  |  |  |  |  |  |  |  |  |  |  |
| V12 | -0.11 | -0.11 | -0.11 | -0.08 | 0.01 | 0.05 | 0.19* | 0.31* | -0.20* | -0.07 | -0.04 | 0.00 | 1.00 |  |  |  |  |  |  |  |  |  |  |  |  |  |  |
| V13 | -0.10 | -0.09 | -0.08 | 0.01 | 0.03 | 0.12* | 0.04 | 0.26* | -0.07 | 0.03 | -0.17* | 0.00 | 0.42* | 1.00 |  |  |  |  |  |  |  |  |  |  |  |  |  |
| V14 | 0.13* | 0.08 | 0.17* | -0.03 | -0.06 | 0.00 | -0.07 | -0.13* | 0.06 | 0.02 | -0.02 | 0.00 | -0.07 | 0.03 | 1.00 |  |  |  |  |  |  |  |  |  |  |  |  |
| V15 | -0.10 | -0.13* | -0.07 | 0.02 | -0.05 | 0.07 | -0.12* | 0.01 | 0.00 | 0.02 | -0.02 | -0.07 | 0.02 | 0.04 | -0.03 | 0.09 | 1.00 |  |  |  |  |  |  |  |  |  |  |
| V16 | 0.02 | 0.07 | 0.06 | 0.00 | 0.02 | 0.03 | -0.08 | 0.10 | -0.09 | -0.02 | -0.18* | -0.04 | 0.05 | 0.19* | 0.04 | -0.35* | -0.07 | 1.00 |  |  |  |  |  |  |  |  |  |
| V17 | 0.02 | 0.00 | 0.00 | 0.18* | 0.02 | -0.16* | -0.04 | 0.05 | -0.13* | -0.08 | -0.15* | 0.09 | 0.01 | -0.08 | -0.15* | -0.09 | 0.05 | -0.04 | 1.00 |  |  |  |  |  |  |  |  |
| V18 | 0.03 | 0.07 | -0.02 | 0.06 | 0.00 | -0.08 | 0.11 | 0.04 | -0.06 | -0.10 | -0.21* | 0.05 | 0.09 | 0.03 | -0.06 | 0.02 | 0.02 | -0.06 | 0.25* | 1.00 |  |  |  |  |  |  |  |
| V19 | 0.13* | 0.08 | 0.14* | -0.08 | 0.07 | -0.09 | 0.02 | -0.20* | 0.08 | 0.02 | -0.05 | 0.02 | -0.06 | -0.10 | 0.05 | -0.03 | -0.03 | 0.02 | 0.14* | 0.07 | 1.00 |  |  |  |  |  |  |
| V20 | -0.12 | -0.2* | -0.13* | 0.04 | 0.07 | 0.11 | -0.05 | 0.02 | -0.02 | -0.05 | 0.09 | 0.06 | 0.04 | -0.01 | -0.02 | 0.07 | 0.04 | -0.12* | -0.01 | -0.06 | -0.07 | 1.00 |  |  |  |  |  |
| V21 | -0.02 | -0.06 | 0.01 | -0.04 | 0.02 | -0.09 | 0.09 | 0.02 | -0.09 | -0.09 | 0.06 | -0.07 | 0.06 | -0.02 | 0.06 | 0.08 | 0.05 | 0.05 | 0.02 | -0.04 | -0.08 | 0.04 | 1.00 |  |  |  |  |
| V22 | -0.07 | -0.2* | -0.06 | -0.01 | 0.01 | -0.05 | 0.02 | -0.05 | 0.02 | -0.09 | 0.21* | -0.03 | -0.03 | -0.12 | -0.10 | 0.14* | -0.04 | -0.12* | 0.06 | -0.02 | 0.07 | 0.14* | 0.13* | 1.00 |  |  |  |
| V23 | 0.07 | -0.01 | 0.06 | 0.06 | 0.04 | -0.03 | 0.10 | -0.11 | 0.00 | -0.01 | 0.14* | 0.05 | 0.02 | -0.16* | 0.05 | 0.12 | -0.14* | -0.08 | -0.01 | 0.01 | -0.06 | 0.19* | 0.19* | 0.34* | 1.00 |  |  |
| V24 | 0.09 | 0.08 | 0.09 | 0.01 | 0.08 | -0.06 | -0.01 | 0.07 | -0.03 | 0.15* | -0.04 | -0.04 | 0.14* | 0.15* | -0.02 | -0.12* | -0.04 | 0.10 | 0.04 | 0.02 | 0.10 | 0.07 | 0.01 | 0.02 | 0.00 | 1.00 |  |
| V25 | -0.01 | 0.08 | 0.03 | 0.04 | -0.05 | -0.01 | -0.03 | 0.05 | 0.01 | -0.09 | -0.02 | 0.06 | -0.09 | 0.03 | -0.03 | -0.07 | 0.02 | 0.04 | 0.20* | 0.11 | -0.01 | -0.04 | -0.08 | -0.08 | -0.03 | -0.02 | 1.00 |

V1, Steps; V2, LPA; V3, MVPA; V4, Age; V5, SitT; V6, Sleep; V7, ST; V8, Gender; V9, BF; V10, Work; V11, Commute type; V12, Consumption of alcohol; V13, Stress; V14, Loneliness; V15, Father’s education; V16, Father’s BMI; V17, Spaces for PE; V18, Extra PA class; V19, Frequency of PE classes; V20, Place to walk; V21, Walk to the bus stop; V22, Paved roads; V23, Lighting; V24, Crime; V25, Commute time to the bus stop.
