# Supplementary material for: Individual, family, school and neighborhood predictors related to different levels of physical activity in adolescents: A cross-sectional study
Source: PLoS One. 2024 Aug 23;19(8):e0304737. doi: 10.1371/journal.pone.0304737 (PMC11343401; doi:10.1371/journal.pone.0304737)
Supplement: Attachment — Submitted filename: Response to Reviewers.docx [file pone.0304737.s004.docx]

Viçosa, Minas Gerais, Brazil. April 7^th^ 2024.

Dear Hsin-Yen Yen, Academic Editor, PLOS ONE,

Firstly, we would like to thank the editor and reviewers for their comments/suggestions/contributions to the manuscript entitled **“Individual, family, school and neighborhood predictors related to different levels of physical activity in adolescents:** **a cross-sectional study”**. We considered that the comments were extremely important and constructive, contributing to improve the quality of the manuscript. It is important to highlight that, for some of the contributions, we tried to better explain our opinions regarding the subject.

We describe each one of the comments along with our answers, which are highlighted in yellow. The changes in the manuscript are also highlighted in yellow.

Hoping to meet the quality requirements of PLOS ONE, we are at your disposal for whatever is necessary.

Kind regards.

**Journal Requirements:**

**When submitting your revision, we need you to address these additional requirements.**

1. Please ensure that your manuscript meets PLOS ONE's style requirements, including those for file naming. The PLOS ONE style templates can be found at 
https://journals.plos.org/plosone/s/file?id=wjVg/PLOSOne_formatting_sample_main_body.pdf and 
https://journals.plos.org/plosone/s/file?id=ba62/PLOSOne_formatting_sample_title_authors_affiliations.pdf

R.	A review was carried out on the manuscript to verify the PLOS ONE style, so modifications were made to the nomenclature referring to the database, which was replaced by “Supporting Information Files”.

2. Note from Emily Chenette, Editor in Chief of PLOS ONE, and Iain Hrynaszkiewicz, Director of Open Research Solutions at PLOS: Did you know that depositing data in a repository is associated with up to a 25% citation advantage (https://doi.org/10.1371/journal.pone.0230416)? If you’ve not already done so, consider depositing your raw data in a repository to ensure your work is read, appreciated and cited by the largest possible audience. You’ll also earn an Accessible Data icon on your published paper if you deposit your data in any participating repository (https://plos.org/open-science/open-data/#accessible-data).

R.	Along with the revised manuscript, the minimum set of underlying data of our study called **“Supporting Information Files”** will be uploaded. Furthermore, the data will be available in the “Public Repository” (https://data.mendeley.com/datasets/gxcs8wzcfb/4).

3. Thank you for stating the following financial disclosure: 
"This study was financed in part by the Coordination for the Improvement of Higher Education Personnel – Brazil (CAPES) – PNPD – CAPES."

Please state what role the funders took in the study.  If the funders had no role, please state: "The funders had no role in study design, data collection and analysis, decision to publish, or preparation of the manuscript." 
If this statement is not correct you must amend it as needed. 
Please include this amended Role of Funder statement in your cover letter; we will change the online submission form on your behalf.

R.	The following excerpts were inserted in the cover letter:

"This study was financed in part by the Coordination for the Improvement of Higher Education Personnel – Brazil (CAPES) – PNPD – CAPES. The funders had no role in study design, data collection and analysis, decision to publish, or preparation of the manuscript."

4. In your Data Availability statement, you have not specified where the minimal data set underlying the results described in your manuscript can be found. PLOS defines a study's minimal data set as the underlying data used to reach the conclusions drawn in the manuscript and any additional data required to replicate the reported study findings in their entirety. All PLOS journals require that the minimal data set be made fully available. For more information about our data policy, please see http://journals.plos.org/plosone/s/data-availability.

"Upon re-submitting your revised manuscript, please upload your study’s minimal underlying data set as either Supporting Information files or to a stable, public repository and include the relevant URLs, DOIs, or accession numbers within your revised cover letter. For a list of acceptable repositories, please see http://journals.plos.org/plosone/s/data-availability#loc-recommended-repositories. Any potentially identifying patient information must be fully anonymized.

Important: If there are ethical or legal restrictions to sharing your data publicly, please explain these restrictions in detail. Please see our guidelines for more information on what we consider unacceptable restrictions to publicly sharing data: http://journals.plos.org/plosone/s/data-availability#loc-unacceptable-data-access-restrictions. Note that it is not acceptable for the authors to be the sole named individuals responsible for ensuring data access.

We will update your Data Availability statement to reflect the information you provide in your cover letter.

R.	Along with the revised manuscript, the minimum set of underlying data of our study called **“Supporting Information Files”** will be uploaded. Furthermore, the data will be available in the “Public Repository” (https://data.mendeley.com/datasets/gxcs8wzcfb/4).

Furthermore, an excerpt mentioning the supporting information file **(Page 12, Lines 294-295)** was mentioned in the manuscript.

“The minimum set of underlying data of our study called “Supporting Information Files” was uploaded. The data will be available in the “Public Repository”.”

5. We notice that your supplementary [Supplementary Table 1] are included in the manuscript file. Please remove them and upload them with the file type 'Supporting Information'. Please ensure that each Supporting Information file has a legend listed in the manuscript after the references list.

R.	The Supplementary Table has been removed from the manuscript and uploaded in a separate file of the 'Supporting Information' file type.

Additionally, each supporting information file has a caption listed in the manuscript after the list of references **(Page 37, Line 803)**.

Furthermore, the text references information from the Supplementary Table 1 **(Page 13, Line 309)**.

6. Please include captions for your Supporting Information files at the end of your manuscript, and update any in-text citations to match accordingly. Please see our Supporting Information guidelines for more information: http://journals.plos.org/plosone/s/supporting-information.

R.	A caption for the “Supporting Information File” has been inserted into the manuscript after the list of references **(Page 37, Line 810)**.

Furthermore, the text mentions the information file and where it is located **(Page 12, Lines 294-295)**.

“The minimum set of underlying data of our study called “Supporting Information Files” was uploaded. The data will be available in the “Public Repository”.”

**Reviewers' comments:**

**Reviewer #1:**

**Dear Authors, I had a real pleasure in reviewing this very interesting manuscript.
In order to improve it I would recommend to:**

- add some statistical results in the abstract - expressed in numbers.

R.	Some statistical results expressed in numbers were inserted in the abstract **(Page 2, Lines 42-49)**.

"Individual and environmental variables were able to respectively predict 64% and 13.6% of adolescents’ participation in LPA. Work (ꞵ_p_ = 0.2322), gender (ꞵ_p_ = -0.1318), commuting to school (ꞵ_p_ = -0.1501), sleep (ꞵ_p_ = -0.1260) and paved roads (ꞵ_p_ = -0.1360) were associated with LPA. It was also observed that individual (59.4%) and environmental (27.4%) variables were able to predict adolescents’ participation in MVPA. Work (ꞵp = 0.1656), commuting to school (ꞵp = 0.1242) and crime (ꞵp = 0.1376, and gender (ꞵp = - 0.3041) and paved roads (ꞵp = -0.1357 were associated with MVPA.”

- add the clear information on WHO recommendation according to the PA in adolescents in introduction.

R.	Information about PA recommendations for adolescents according to the WHO was added to the text **(Page 3, Lines 74-77)**.

“According to the World Health Organization (WHO), adolescents should practice, on average, at least 60 minutes of MVPA per day and incorporate at least 3 days per week of vigorous-intensity aerobic activities, as well as those that strengthen muscles and bones [10]”.

- add the information on VPA as one of recommended by WHO existing measurements of levels of PA in adolescent with information why you haven’t decided to check the level of VPA in your research.

R.	Please, note that according to WHO guidelines, adolescents are advised to engage in a minimum average of 60min/day of moderate-to-vigorous PA, rather than a minimum requirement specifically for VPA (with an emphasis on a combination of both moderate and vigorous PA). With this in mind, our focus was on investigating MVPA, and not MPA and VPA separately.

In addition, the WHO guidelines advocate for the principle that “every movement counts”, encouraging, when possible, the reduction of sedentary behaviour, by substituting it with LPA. Taking this into account, in addition with the fact that available evidence supports the health benefits of LPA (and not only MVPA), we also included LPA in the analysis.

In summary, our decision to investigate MVPA and LPA aligns with WHO guidelines and current scientific literature/evidence. We hope the reviewer understands our decision.

Information regarding the VPA recommendations was added to the text when we responded to the previous question, regarding the WHO recommendations on PA for adolescents **(Page 3, Lines 74-77)**.

“According to the World Health Organization (WHO), adolescents should practice, on average, at least 60 minutes of MVPA per day and incorporate at least 3 days per week of vigorous-intensity aerobic activities, as well as those that strengthen muscles and bones [10]”.

- add definitions of LPA and MVPA - in the methods section.

R.	The definitions of LPA and MVPA have been inserted into the text **(Page 6; Lines 139-141)**.

“ActiGraph GT3X accelerometer was used to monitor the time spent in light PA (LPA) and moderate-to-vigorous PA (MVPA) (min.day^-1^), and the sleep/wake time (hours.day^-1^).”

**Reviewer**

**Reviewer #2: Dear Authors, thank you for the opportunity to review the paper titled Individual, family, school and neighborhood predictors related to different levels of physical activity in adolescents. Here are my comments.**

**Major Concerns**

1. High schools were randomly sampled. If I understand correctly, all students of those high schools were asked to participate. However, because of the sampling of the high schools it is a multistage sample anyway. Because of this sampling method clustering is introduced in the data. Therefore, the school level should have been taken into account in the analyses: multilevel analysis.

R.	There are some assumptions that must be taken into account for the adoption of multilevel analysis: the random selection of components at each level; a minimum number of clusters at level 2 and above; and a minimum number of subjects within each cluster (Austin, 2010; Bell et al., 2010; Austin e Leckie, 2018).

The random selection of components at each level aims to prevent selection bias in the results. In our study, subjects (level 1) were randomly selected, as described in the methods section. However, the same procedure was not applied during the school selection process, i.e., they were not chosen randomly, but rather because they were the only ones in the city offering public secondary education. And based on this selection process, including these schools in the second level of multilevel analysis would violate one of the assumptions of the analysis.

Furthermore, regarding the number of observations within each level, while different authors propose varying numbers of observations (Austin 2010; Bell et al., 2010; Austin e Leckie, 2018), it is generally recommended that this number must be, at least, greater than 10 (Austin, 2010), (some authors suggest at least 20 (Austin e Leckie, 2018), or even 30 (Bell et al., 2010)). This is because it is the minimum required for accurately estimating the variance component at each level. In our study, we only sampled six schools, which would not have provided a sufficient number of clusters for conducting the multilevel analysis.

However, we were cognizant of the role of the school on the results, and with this in mind we performed the analysis adjusting for the cluster (i.e., school), as has been done in previous studies where the multilevel structure of the data was presented, but the multilevel analysis could not be conducted due to failure to meet the required assumptions.

2. Perceived school environment predictor are included in the questionnaire, while multiple students per school were asked to participate. These predictors are more similar in students attending the same school than in students attending different schools. This is another reason to include the school level in the analyses. In addition, one might hypothesize that perceived school environment predictor are different in each class; therewith introducing clustering at the class-level, too.

R.	We have previously outlined the reasons for not using the multilevel analysis, considering school as a level. Since we were unable to conduct the analysis considering school as the second-level, it makes not possible to include the class as a third-level.

Furthermore, within each school, students were sampled from the same class, which means the absence of within-school differences regarding class.

3. On top of that, there is the neighborhood level. Neighborhood predictors are also included in the study. I assume that students attending the same school also live in a limited number of neighborhoods. This introduces clustering per neighborhood. In addition, depending on the distance between the high schools, students living in one neighborhood can attend different schools. If so, data do not have a normal multilevel structure, but a cross-level structure. When in the analysis of multilevel data clustering is ignored, standard errors and significances are not correct.

R.	The sample comes from 39 different neighbourhoods. However, in 51% of these neighbourhoods there were five or fewer adolescents residing. According to Austin (2010), when there are five or fewer observations per cluster, the estimates of the variance (both, dependent variable and standard error) are prone to be inefficient.

Once again, considering the assumptions of the multilevel analysis (Austin, 2010; Bell et al., 2010; Austin e Leckie, 2018), our data does not meet the requirements (minimum number of clusters, and minimum number of observations in each cluster), which would lead to an inadequate estimates of the variance components, standard errors, confidence intervals, and the overall model. Therefore, the adoption of the Ordinary Least Squares (OLS) estimates was our choice, as it allowed us to estimate the coefficients of the final model, with the adjustment for clustering, without diminishing the explanatory power of the model.

4. Please amend the title to include the research question and study design.

R. The title was changed to include the study design (Page 1; Lines 4-5).

“Individual, family, school and neighborhood predictors related to different levels of physical activity in adolescents: a cross-sectional study.”

**References**

Austin P. C., Leckie G. The effect of number of clusters and cluster size on statistical power and Type I error rates when testing random effects variance components in multilevel linear and logistic regression models. Journal of Statistical Computation and Simulation, 2018, 88:16, 3151-3163. DOI: 10.1080/00949655.2018.1504945.

Austin PC. Estimating multilevel logistic regression models when the number of clusters is low: a comparison of different statistical software procedures. Int J Biostat. 2010, 22;6(1). DOI: 10.2202/1557-4679.1195.

Bell B. A, Morgan G. B., Kromrey J. D., Ferron J. M. The Impact of Small Cluster Size on Multilevel Models: A Monte Carlo Examination of Two-Level Models with Binary and Continuous Predictors. Section on Survey Research Methods – JSM 2010.

<http://www.asasrms.org/Proceedings/y2010/Files/308112_60089.pdf>

Romanzini M., Petroski E.L, Ohara D., et al. Calibration of ActiGraph GT3X, Actical and RT3 accelerometers in adolescents. Eur J Sport Sci. 2014;14(1):91–9. doi:10.1080/17461391.2012.732614.

Wooldridge, J.M. (2009). Introductory Econometrics: A Modern Approach. 4th Edition, South-Western College Publishing, Cengage Learning, Boston.
